# Study of historical evacuation drill data combining regression analysis and dimensionless numbers

**DOI:** 10.1371/journal.pone.0232203

**Published:** 2020-05-01

**Authors:** Maria D. Miñambres, Diego R. Llanos, Angel M. Gento

**Affiliations:** 1 Servicio de Prevención de Riesgos Laborales, Universidad de Valladolid, Valladolid, Spain; 2 Dpto. de Informática, Universidad de Valladolid, Valladolid, Spain; 3 Dpto. de Organización de Empresas y Comercialización e Investigación de Mercados, Universidad de Valladolid, Valladolid, Spain; Universitat de Valencia, SPAIN

## Abstract

The time needed to evacuate a building depends on many factors. Some are related to people’s behavior, while others are related to the physical characteristics of the building. This paper analyzes the historical data of 47 evacuation drills in 15 different university buildings, both academic and residential, involving more than 19 000 persons. We propose the study of the data presented using a dimensionless analysis and statistical regression in order to give a prediction of the ratio between exit time and the number of people evacuated. The results obtained show that this approach could be a useful tool for comparing buildings of this type, and that it represents a promising research topic which can also be extended to other types of buildings.

## 1 Introduction

To implement the evacuation of an entire facility requires a great organizational effort, where all the staff is involved. It is crucial to be very careful so as to avoid generating confusion or demotivation among staff or public. In addition, it is not always possible to have enough resources to gather all the data that is desirable. When several evacuation drills have been carried out in a building, safety and security staff usually compare results, but these results are not often comparable to results from other buildings.

This paper analyzes the historical results of 47 evacuation drills in 15 different university buildings, both academic and residential, involving more than 19 000 persons. We propose the study of the data using a dimensionless analysis and statistical regression in order to give a prediction of the time ratio between exit time and the number of people evacuated. As far as we know, our approach has not been used before in the field of evacuation drills.

The time used in the study is time elapsed from the evacuation alarm to the end of the evacuation. This time, that we call *exit time*, is be the sum of the pre-evacuation time (that is, the time elapsed from the moment when the alarm sounds and when the occupants decide to start the evacuation) plus the movement time. With the help of the data related to the characteristics of the evacuated buildings, we have calculated a dimensioness parameter associated to each building, that we call the *Characterization of building evacuation (CBE)*. Given the CBE for a particular building and the number of people occupying it, we are able to calculate an estimated exit time. Comparing this estimated exit time with the real values obtained in evacuation drills, more informed decisions on whether to invest in more training and/or preventive culture of the occupants or to invest in structural improvements of the buildings can be taken.

To build this study, we have analyzed the historical information collected by the University of Valladolid over the last decade from a total of 47 evacuation drills of 15 university buildings, both academic and residential, invoving 19 198 occupants and 688 external observers. Most of this information has been used to build the study introduced in this work, while the remaining evacuation drills have been used to validate the obtained results.

We are aware that this sudy cannot be used directly to compare buildings of other types. Instead, we see this approach as a promising method to compare exit times of buildings that share the same or similar characteristics.

The paper is organized as follows. Section 2 discusses some related work. Section 3 describes the techniques used and the fundamentals our model is based upon. Section 4 presents both the structural characteristics of the buildings used to feed the study, and the results of the evacuation drills conducted in them. Section 5 shows the details of our study, including the definition of the Characterization of Building Evacuation (CBE), the regression formula obtained from the evacuation drills conducted, and the associated formula for the estimated exit time, $\hat{Te}$. Section 6 compares and discusses the differences between the average exit time obtained in evacuation drills for each of the buildings considered, and the value for $\hat{\text{Te}}$ returned by the model. Section 7 applies the model to a different set of buildings and discusses the results. Finally, Sect. 8 concludes our work.

## 2 Related work

Published work on different mathematical models of behavior in different emergency situations has increased enormously in recent years. Emergency situations include fire, e.g., e.g., [1], terrorist attacks [2], natural disasters [3], specific behaviors such as evacuation processes in areas with internal obstacles [4], etc. Regarding the field of human behavior in fire, Kuligowski [5] gathered all the available data, studies and research at that moment, including evacuation dynamics, timing for certain aspects of building evacuations, analysis of the characteristic movements of vulnerable population, and modelling of evacuation movements. More recently, Ronchi et al. [6] summarized recent findings in the field of fire evacuation modelling of different topics within research disciplines outside fire safety engineering, including Applied Mathematics, and Dynamic Simulation and Biomechanics, aiming to study the feasibility of development and application of modelling methods based on these fields and to discuss their implementation strengths and limitations.

Related work also includes results regarding different surroundings: Buildings of various sizes, from skyscrapers to multi-story office buildings, e.g., [7]; public buildings such as train stations or underground, e.g., [8]; tunnels [9, 10], etc. In most of them, where behavior is modelled to predict evacuation time [11], they use algorithms that require the layout of escape routes in buildings, together with models that can be continuous or discretized in a grid [12]. However, as Lovreglio *et al.* [13] pointed out, many of these models are based on the comparison of a single experimental data-set with simulation results. The associated algorithms contain complex methods of calculation for assessing crowd flow, based on equations. Essentially, this consists of calculating the number of people in a specific area, in order to obtain the density, then deriving the speed and flow rate of the crowd. Algorithms, also take into account merging traffic flows, and the resulting changes in density and flow rate. Initially, these tedious calculations were carried out manually, as for example with Predtechenskii and Milinskii equations (as appear in [14]) where the building is segmented into a network of discrete areas, linked with doorway ‘nodes’ of specific widths. Haghani and Sarvi [15] developed an extensive compilation of all empirical studies carried out to date, both with people and animals, concluding that there is a lack of unification to allow studies to be comparable and reproducible.

In this work, we present the information on evacuation drills conducted by the University of Valladolid during the last decade in different university buildings, both academic and residential, together with the buildings’ main characteristics. We also propose the study of the data using a dimensionless analysis and statistical regression in order to give an expected value of the ratio between exit time and the number of people evacuated. We are aware that our procedure for estimating the evacuation time is simpler than other well-established methods described above. However, the main advantage of our proposal is that it facilitates the comparison between the evacuations of different buildings with a method that requires some simple calculations that can be carried out without a great investment.

## 3 Our proposal

Dimensional analysis is a method for reducing complex physical problems to their simplest (most economical) forms prior to quantitative analysis or experimental investigation [16]. Dimensional analysis has been used in different science areas, in some of them for a long time [17]: Chemistry, mathematics, physics (electricity and magnetism, sound, fluid mechanics, etc.), and, more recently, in economics. The Vaschy-Buckingham *π* theorem [18, 19] provides a method for calculating sets of dimensionless parameters from the given variables, even if the shape of the equation is still unknown and the choice of dimensionless parameters is not unique. It is necessary that the choice of variables has a physical meaning.

Regression analysis is a conceptually simple method for investigating functional relationships among variables, dependent (target) and independent (predictor) variable(s). There are several methods of regression analysis and it is still an area of active research. These predictive modeling techniques have numerous areas of application, such as business, economics, education, engineering, finance, medicine, meteorology, psychology, sociology, etc.

Dimensional analysis and regression analysis have not been used together in evacuation studies but they have been used together in other areas. Vignaux and Scott [20] show that, with dimensional analysis, factors without dimension can be used as variables of the regression. As a result, fewer values than the originals are obtained, often offering a more appropriate interpretation. They showed practical examples in the area of mechanical engineering. Other practical examples can be found in the work by Shen *et al.* [21]. The model proposed in this paper has the same benefits and limitations introduced in the work of Vignaux and Scott [20].

The aim of this work is to analyze the historical results of many evacuation drills in several different university buildings, both academic and residential. Using this data, we carry out a study that estimates the exit time that can be expected from a particular building, based on both the architectural characteristics of the building and the data collected from previous evacuation drills of this building and others of the same type.

According to Cuesta *et al.* [22], exit time is influenced by: The layout and distance to be travelled by people; bottleneck points; added times relative to different circumstances and choices; and speed of movement of the people. We consider “exit time” to be the interval between the moment the sirens start to sound until the moment when no more people come out of the building, the moment that consider to be the end of the evacuation. This interval includes pre-evacuation and movement times.

In order to manage the characteristics of the buildings, we propose the use of a coefficient called “Characterization of Building Evacuations” (CBE). This value is obtained with the help of a formula that depends on a reduced number of measurable parameters of the building. This formula substitutes the need for both a mathematical modelling of all possible routes of evacuation of a building, and a computer simulation of the equations of human behavior based on the planned routes. Our idea was inspired by dimensional analysis, where, instead of solving complex equations, calculations are simplified thanks to the use of previously calculated, dimensionless numbers. For example, in fluid mechanics, these coefficients are calculated for different fluids, in different modes of behavior, and working in a laminar or turbulent regime. As long as it is not proven that variables of human behavior can be considered in the same way as physical magnitudes, the *π* theorem of Vaschy-Buckingham [18, 19] cannot be applied directly to find the equation that models the behavior of people in the evacuation, which is the product of a regression analysis [20].

Given the CBE dimensionless number (that depends on the architectural characteristics of the building) and the occupation of a given building (the number of people inside it at the time of the evacuation), an estimated time needed for the evacuation can be calculated (exit time). This estimated time can be compared with the real time obtained in evacuation drills.

Our dimensionless number considers the following points:

- The distance to be travelled by people. It depends on the design of the building: In general, the bigger the surface, the bigger the average distance to be travelled and the greater the evacuation difficulty. We are aware that, for the same surface, the distance is also affected by other circumstances, such as number of exits, number of floors, number of staircases, or different surface geometries.
- Number of exits: The fewer the number of exits, the greater the possibility of delays and the greater the evacuation difficulty.
- Width of exits: The smaller the width of the exits, the greater the possibility of delays and the greater the evacuation difficulty.
- Points of exit from each floor to the staircases: The larger the number of floors, the greater the possibility of conglomerations at the exits or in the floors and the longer the delays, making the evacuation more difficult. Besides this, the fewer the number of staircases leading to these floors, the greater the possibility of slowing down the evacuation and the greater the evacuation difficulty.

In the following sections, each building is characterized through: Surface area, number of floors, number of staircases, number of exits, and width of the exits. Other parameters could be considered as well, such as staircase widths (that must have a minimum width in accordance with the Spanish regulations of health and safety at work). Our analysis stopped at this point because we have found that the use of the chosen parameters allows the of a formula that fits very well with the experimental data, as we will see in the following sections.

The aim of this study is not to yield 100% accurate results, but to offer a useful tool to help companies and organizations to know what to expect from the exit times and where they should prioritize their interventions, either by reducing the difficulty of evacuating a building or by training and sensitizing the employees/occupants of the building. Besides this, the proposed formula for the dimensionless number has been derived from data related to university buildings with the characteristics that are described in this paper. Future works should check if this formula is valid for the same type of buildings in non-university contexts. We think that it could be suitable as long as most of people in those contexts do not have physical, psychic or social impediments that limit their ability to evacuate, as it happens in university buildings. In the same way, it would be necessary to test if this dimensionless number formula is valid for another type of buildings with different physical characteristics (for example, with greater surface, many more floors, etc.) We believe that the this approach is an interesting path in better understand the relationship between building characteristics and exit times.

## 4 Data collection

The University of Valladolid have several buildings in four cities with different sizes, uses and characteristics. Evacuation drills are held periodically as part as of the routine of the university. The drills have been designed for the training of the staff and users, as well as to detect possible problems in the evacuation or detection of emergencies. The process of conducting the drills includes the collection of data and the completion of a report. This report includes exit times, the observers who have participated and where they are located, events and issues to improve. Reports are sent to leaders and unions. In addition, exit times are sent to Deans, Directors and to every employee in the building in the following hours of the drill. These data of evacuation and exit times are those that have been used to perform the present study. The observers were mostly university students who did practical exercises of their university studies with subjects related to emergencies and evacuations.

This study analyzes the historical data of evacuation drills at the University of Valladolid. No evacuation drill has been specifically conducted to carry out this study. A total of 688 persons observed 646 points in 47 drills, with a total of 19 198 people evacuated in these drills. Some points had two observers. Every volunteer was an observer in two or more evacuation drills. Observers were trained for their task in order to collect impartial observations of the behavior of the evacuated occupants considered as a group. The qualitative information collected in the questionnaires and in the post-drill meetings has been used to validated the coherence of the data results of the model, as explained in Sect. 6. Just before each drill, a meeting was held with all the observers to make sure they knew what and where to do, and how not to interfere with the evacuation.

In each drill, observers are placed at the exits of a building, at the meeting point outside the building, always in places that do not interfere with the development of the evacuation. The observers are provided with questionnaires where they can record the behavior of people in the evacuation, incidents, comments, and observations, as well as the timestamp of each of the possible milestones in the evacuation (when the first/last evacuated person passes through a certain point, etc.). The start time (activation of the evacuation sirens) is used to calibrate the rest of the timestamps collected by each observer. After the drill, the observers attend a second meeting, together with maintenance personnel of the building, to collect together the issues observed, as well as possible incidents during the course of the evacuation.

The historical data available have been divided into two sets. In order to carry out this study, we have used the data of 44 evacuation drills from 12 different buildings. There are between two and five drills from each of the buildings considered. The more evacuation drills from the same building, the less uncertainty concerning human behavior [13] and the more representative the results. However, the organizational costs of an evacuation drill are high, so it is not feasible to organize additional evacuation drills to have more data available. The results of the model obtained were then used to analyze individual drills of three additional buildings (as will be seen in Sect. 7).

At every evacuation drill, the exit time and the number of people evacuated were measured. Regarding time, we measured the time needed to perform the evacuation, considered from the start of the sirens until the evacuation is considered finished, as well as other intermediate times in order to check the process. The time was recorded in whole minutes, having weighed up the pros and cons of recording the data in seconds. Minutes were chosen over seconds because of the problems of getting observers to record exact times in seconds, mostly due to the need to write the data while continuing with the observation, and the problem of exact calibration of clocks.

Regarding the approximate number of people who have evacuated the building (Np), this number is the sum of all the estimations of people leaving the building using each one of the exits towards the outside, as collected visually by the observers. The figures offered in our measurements are rounded in most cases because the measures are not 100% reliable, due to the fact that observers could pay attention to other qualitative information at that moment. In quantities below 100 persons, the count is more accurate.

To build the CBE formula, we have used the following building parameters:

- The average number of exits per floor that can be used as evacuation exits (*Ea*).
- The sum of the widths of the exits towards the outside of the building (*Me*).
- Number of different staircases that can be used as evacuation paths (*St*).
- Number of floors that are usually occupied (*F*). The floors occupied occasionally for maintenance issues are not included, such as the rooftop of the building if it is only accessed for maintenance operations, or the basement if there are only technical rooms exclusively for maintenance operations.
- Total surface of the parts of the building usually occupied (*Sf*). In the case of discrepancies among building projects, building maps, and legal registers, the latter are used.

It is interesting to note that the Spanish regulations establish a maximum Theoretical Occupancy (TO) for a given building, taking into account its surface and the type of use for the building. The Theoretical Occupancy is used by building designers to adequately calculate the length and width of the evacuation routes. The values of TO for each building considered are depicted in Table 1 Comparing the occupation of the evacuation day (Np) with the Theoretical Occupancy (TO), it can be determined whether the building had high occupation during that day. This, in turn, serves as an indicator of whether the routes might be saturated, and whether the people in the evacuation drill might have felt pressure or stress to evacuate the building.

**Table 1:** Data used to build our model: Buildings with more than one evacuation drill.

Drill	Sf	F	St	E	Me	TO	Np	Te
EII-SPC 2013-11	14 683	4	3	3	10	3 494	700	9
EII-SPC 2015-10	14 683	4	3	3	10	3 494	700	9
EII-SPC 2016-11	14 683	4	3	3	10	3 494	700	9
EII-SPC 2017-11	14 683	4	3	3	10	3 494	800	7
EII-SPC 2018-11	14 683	4	3	3	10	3 494	500	9
EII-SFM 2015-05	13 185	6	6	4	6.8	3 021	500	5
EII-SFM 2016-03	13 185	6	6	4	6.8	3 021	500	6
EII-SFM 2017-03	13 185	6	6	4	6.8	3 021	800	5
EII-SFM 2018-05	13 185	6	6	4	6.8	3 021	350	4
FC 2013-04	15 107	4	6	4	8.1	1 470	175	9
FC 2016-11	15 107	4	6	4	8.1	1 470	175	8
FC 2017-11	15 107	4	6	4	8.1	1 470	200	6
FC 2018-11	15 107	4	6	4	8.1	1 470	180	5
AFC 2013-04	11 166	4	7	7	11.23	3 131	225	8
AFC 2016-11	11 166	4	7	7	11.23	3 131	600	9
AFC 2017-11	11 166	4	7	7	11.23	3 131	900	5
AFC 2018-11	11 166	4	7	7	11.23	3 131	900	6
ETIC 2010-03	21 009	3	5	8	14.4	3 169	700	6
ETIC 2011-04	21 009	3	5	8	14.4	3 169	700	10
ETIC 2016-03	21 009	3	5	8	14.4	3 169	700	7
ETIC 2017-03	21 009	3	5	8	14.4	3 169	700	6
ETIC 2018-05	21 009	3	5	8	14.4	3 169	500	6
FFIA 2015-05	21 709	6	6	5	25	3 029	1 000	9
FFIA 2015-11	21 709	6	6	5	25	3 029	1 000	12
FFIA 2017-05	21 709	6	6	5	25	3 029	500	6
FFIA 2018-05	21 709	6	6	5	25	3 029	1 000	7
AVIII 2010	22 726	9	4	10	14	3 269	200	8
AVIII 2015-05	22 726	9	4	10	14	3 269	130	7
AVIII 2015-10	22 726	9	4	10	14	3 269	130	10
AVIII 2017-11	22 726	9	4	10	14	3 269	150	12
AVIII 2018-11	22 726	9	4	10	14	3 269	300	8
CMSCF 2016-05	6 514	8	3	4	3.4	145	15	8
CMSCF 2016-11	6 514	8	3	4	3.4	145	50	6
CMSCF 2017-11	6 514	8	3	4	3.4	145	55	4
CMSCF 2018-11	6 514	8	3	4	3.4	145	40	5
BRS 2013-04	2 155	2	1	2	2.01	362	80	4
BRS 2014	2 155	2	1	2	2.01	362	80	4
ETSA 2016-03	13 605	5	8	4	14.8	2 615	350	5
ETSA 2018-05	13 605	5	8	4	14.8	2 615	250	5
CMSCM 2017-05	4 057	5	2	3	4.1	579	50	6
CMSCM 2017-11	4 057	5	2	3	4.1	579	50	4
CMSCM 2018-11	4 057	5	2	3	4.1	579	31	3
LUCIA 2018-05	5 321	3	2	2	3.4	476	64	4
LUCIA 2018-11	5 321	3	2	2	3.4	476	43	3

It is also important to note that all the buildings meet at least the minimum safety conditions stated by the EU regulations with respect to emergencies in workplaces, including evacuation signage.

In summary, our model is valid for buildings similar to those considered in our study, that is: Buildings that accomplish EU regulations for buildings with respect to safety in workplaces; buildings of similar sizes to the ones used in our study, both in terms of building surface and number of floors; and buildings with similar uses, that is, we do not consider industrial working places.

Table 1 summarizes all the data used for this study, gathered as described above. This table shows the data for buildings with two or more evacuation procedures used to derive the model.

## 5 The study

Based on the aforementioned parameters and how we believe they influence the difficulty of evacuation, we propose the following formula for CBE:

$$\begin{array}{c} CBE=\frac{\sqrt{Sf}}{Me}*\frac{F}{Ea} \end{array}$$ (1)

where *Sf* is the surface of the building, *Me* is the sum of the widths of the building exits in meters, *F* is the number of usually occupied floors (as described in the previous section), and *Ea* is the average number of exits per floor, including floor exits towards the staircases and floor exits towards the outside.

*Ea* is obtained as follows:

$$\begin{array}{c} Ea=\frac{St*(F-1)+E}{F} \end{array}$$ (2)

where E is the number of exits from the building towards the outside, and St is the number of staircases going to the exit or to a main evacuation route.

Table 2 shows the value of CBE for all the buildings where more than one drill took place. Average values for the number of people evacuated (*ANp*) and average exit times (*ATe*) for each building have been used to perform the corresponding regression analysis. CBE depends only on the structural parameters of the building that influence evacuation time, so it is taken as the independent variable. In this way, the analysis separates the structural issues that influence evacuation times from issues related to human behavior.

**Table 2:** Average number of people and exit times, and CBE for the considered buildings.

Drill	Avg. Np (ANp)	Avg. Te (ATe)	(ATe/ANp)*100	CBE
EII-SPC	680	8.6	1.25	16.16
EII-SFM	537.5	5	0.93	17.88
FCMD	182.5	7	3.84	11.04
AFC	656.25	7	1.07	5.38
ETIC	660	7	1.06	5.03
FFia	875	8.5	0.97	6.06
AVIII	182	9	4.95	20.77
BRS	80	4	5.00	30.79
CMSCF	40	5.75	14.38	60.77
CMSCM	50	4.33	8.67	35.31
ETSA	300	5	1.67	5.47
LUCIA	53.5	3.5	6.54	32.18

**Fig 1:**
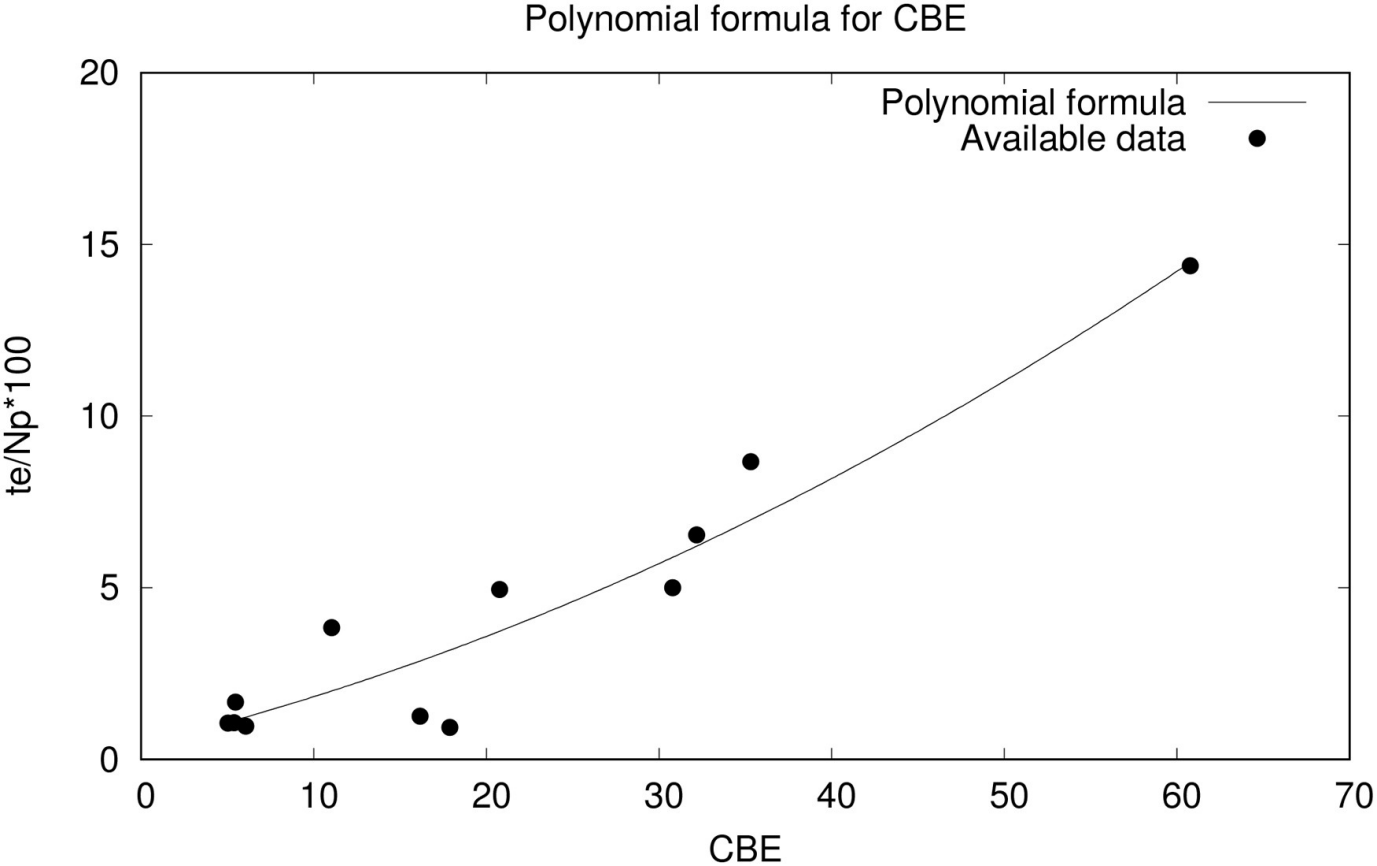
Polynomial formula for CBE based on available data.

In order to use CBE to predict the exit time for other buildings, it is necessary to derive a formula that, for a given CBE, returns the value for (ATe/ANp)*100, which in turn can be used to estimate the average time (ATe) needed to evacuate a building, for a certain number of persons (ANp). We have chosen a polynomial formula for this purpose. Adjusting the formula to the data shown in Table 2, we reach the following expression (see also Fig 1):

$$\begin{array}{c} f\left(x\right)=0.0018x^{2}+0.1218x+0.4289 \end{array}$$ (3)

The value of *R*^2^ for this expression, using the available data, is 0.911. We consider this value good enough to predict the exit time with respect to the population of the building and its architectural characteristics. (Using other families of regression formulas returns a lower *R*^2^ value.) This formula can be used to get estimations of the exit time, $\hat{Te}$:

$$\begin{array}{c} \hat{Te}=\frac{Np*f(CBE)}{100} \end{array}$$ (4)

In the following sections, we analyze the coherence of the estimations returned by our model in comparison with the real qualitative information, for these and other buildings.

## 6 Goodness of the study

In this section, we compare and discuss the differences between the average exit time (ATe) for each of the buildings considered and the value for $\hat{\text{Te}}$ returned by the model.

Table 3 shows both the ATe and $\hat{\text{Te}}$ for each building. As we said in Sect. 3 having a good coefficient of determination *R*^2^ is not enough. In order to check the coherence of the proposed model, we can make the following observations:

EII-SPC and EII-SFM: Evacuation drills in both buildings were faster than the time indicated by the value of their CBE and number of occupants. The reasons may be related to the particular involvement of the center’s management of both buildings, as well as the interest and sensitivity shown by both employees and students, partly because safety and protection issues are taught there as part of their academic programs.
FCMD: Evacuation drills in this building were slower than expected according to its CBE. It is consistent with a known difficulty (admitted by the Deans of this academic center) regarding the attitude of many employees of this particular center, who consider the evacuation drills an activity that should not be carried out during working hours and/or during lectures.
ETIC and AFC: Both centers were evacuated in a time very similar to the time estimated by our model. If we examine all the evacuation drills, we can see an evolution in their behavior that is consistent with the actions for the improvement of the preventive culture.
FFIA: People at this building carried out the evacuation in less time than the time expected by our model. The center is known for being particularly involved in this activity. We can see an evolution in their behavior similar to the ETIC and AFC but with a better improvement in the results.
AVIII: This is a student residence, with some spaces hosting other activities. Different directors are responsible of each activity, with different levels of involvement in conducting drills. Almost all drills were done early in the morning, so it does not seem strange that the residents who were still in their rooms needed more time to evacuate the building.
BRS: This is a historic and small building in comparison with the rest of the premises. It is used as a library where librarians are actively involved in risk management activities and have a very effective organization to evacuate students and researchers. These reasons can explain why the effective exit time was shorter than the value predicted by our model.
ETSA: People in this building performed the evacuation in longer times than expected according to our model, not improving their behavior in subsequent evacuation drills. The particular attitude of the occupants of this building towards this subject could have affected the evacuation drills, because they do not complain about the activity but neither do they show active participation in this matter.
CMSCF and CMSCM: Both buildings have the same management, which is very involved with security and safety issues. Almost all the students have attended a seminar on how to act in case of emergency. The results from CMSCM diverges more from the model than those from CMSCF, although it can be seen that the estimated time for the former building is the same exit time as the last evacuation drill of the center. CMSCF behaves as the model predicts.
LUCIA: The model makes a good prediction in this case. It is a building with laboratories, spaces and facilities dedicated to research. Their occupants are researchers with experience in evacuation drills of other buildings.

**Table 3:** Evaluation of the CBE model with respect to average exit times.

Drill	ATe	$\hat{Te}$
EII-SPC	8.6	19.49
EII-SFM	5	17.1
FCMD	7	3.64
AFC	7	7.45
ETIC	7	7.18
FFia	8.5	10.79
AVIII	9	6.8
BRS	4	4.71
CMSCF	5.75	5.79
CMSCM	4.33	3.49
ETSA	5	3.45
LUCIA	3.5	3.32

## 7 Applying the model to other buildings

The proposed model offers a prediction for groups of people of similar characteristics to our population, and for buildings in the range of size and characteristics of the buildings described in this paper. Comparing the prediction value with the experimental value, we can see whether the people evacuated show a better or worse behavior than the values returned by the model. In the latter case, it would be wiser to invest in improving their knowledge and safety culture, in order to reduce the exit time. If they behave better, then it would be better to invest in improving the building’s conditions to further reduce the exit time.

In order to show the effectiveness of our model, we apply it to examine the results of three different buildings where a single evacuation drill has been carried out. These drills and buildings were not used to build the model, because we believe that one single evacuation drill is not enough for the results to be representative. We use the evacuation drills shown in Table 4.

**Table 4:** Data for a set of buildings with one evacuation drill.

Drill	Sf	F	St	E	Me	TO	Np	Te
AETSA 2017-03	3 700	2	2	2	3.2	657	350	9
EMZ1 2013-05	10 810	4	14	14	19.65	3 469	975	9
ETSIAP 2011-03	7 633	3	3	2	6	1 961	100	5

We have followed the same steps as those described in Sect. 4 for the observation of the evacuation drills. In this case, a total of 40 people observed 40 points in these three drills, with a total of 1 425 people evacuated. The calculation of their CBE and the comparison with the estimated exit time can be found in Table 5. Based on these results, we can make the following observations:

**Table 5:** Evaluation of the CBE model with respect to exit times of buildings with one drill. For these buildings, ATe becomes Te (only one datum).

Drill	Te	$\hat{Te}$	CBE
AETSA 2017-03	9	11.88	19.01
EMZ1 2013-05	9	6.02	1.51
ETSIAP 2011-03	5	2.91	16.38

AETSA: The occupants clearly behave better than the prediction of the model. The context is interesting to analyze. AETSA is a building used for delivering lectures of the ETSA school, because ETSA does not have enough classrooms. It was the first evacuation drill for AETSA, but many people have participated in previous evacuation drills of ETSA. Many of them know that ETSA is bigger than AETSA and that ETSA was evacuated in five minutes. This could be an anchoring effect [23] that has a positive impact in this case, because they do not know that, according our model, AETSA is more difficult to evacuate, being even smaller. In this case, our model suggests that, if we wanted to improve the exit time in this building, then it would be better to invest in the building itself.
EMZ1: Considering the building’s characteristics, the exit time forecast seems to be reasonable. Therefore, according to the model, the occupants should improve their behavior. In fact, it was their first evacuation drill and the only drill organized by the Health and Safety Service of the University. This building is one of the two buildings analyzed that are located in a different city, and, for organizational and historic reasons, it is more difficult to involve people in procedures organized remotely from the University headquarters. In this case, the model confirms our impression: If we wanted to improve the exit time in this building, it would be better to invest in improving safety culture.
ETSIAP: As happened with the previous building, the exit time forecast is reasonable to achieve and, according to the model, the occupants’ behavior still has room for improvement. It was also their first evacuation drill, and ETSIAP is the other building located in a different city, but not in the same city as EMZ1. The process to involve people in procedures organized remotely from Valladolid is also difficult.

In short, we have found that all the results have a qualitative coherence with respect to the collected data. The quantitative deviations observed between real and predicted results are consistent with the different behavior patterns of each group of people in each building.

## 8 Conclusions and future work

We conducted an innovative regression analysis of historical data from evacuation drills of some buildings in our university campuses. The regression analysis has been drawn by analyzing the relationship between the exit time vs. the number of people evacuated in the evacuation drill for each building, a value that we relate to a dimensionless parameter that we have called the Characterization of Building Evacuations (CBE). The CBE can be calculated using simple structural parameters of the building, making the model easy to use. CBE is a dimensionless value that only depends on the characteristics of the building and is completely independent of the behavior of the people involved. We have proposed a polynomial formula that fits the values with sufficient goodness, complementing the study with an exhaustive qualitative analysis that leads to consistent results.

The main added values of our model are the following: First, given a certain building whose CBE number can be calculated, and given the number of people in an evacuation drill, our model returns an estimated exit time. The relationship between the estimated and real times can be used to guide activities to improve the latter. If the estimated time is shorter than the measured time, it means that people behave worse than expected. This would suggest that it might be better to invest in improving people’s behavior than investing in improving facilities to ease the evacuations. On the contrary, when the estimated time is longer than the measured time, it would suggest that it might be better to invest in the facilities to further ease the evacuation.

Second, this form of study can be used to analyze and compare the dimensionless number of other buildings of a similar size and characteristics (number of floors betweeen 2 and 9, surface between 2 000 and 23 000 square meters), These characteristics are easily met by most of buildings designed for educational purposes, as well as office buildings and student residences. In fact, our approach is not limited to buildings of these characteristics: It can be applied to other types of buildings as well, just by collecting the indicated data, making a few calculations and comparing the results with the results obtained in their own evacuation drills, without making a significant investment.

It should be taken into account that the model was designed to be used with buildings that satisfy the legal minimums established in European regulations on safety in workplaces (regarding signage, expedited evacuation routes, etc.), and also with a population that does not, in general, present big disabilities.

Our future work includes improving the estimations given by this proposal, thus improving the strategic decisions taken about evacuation subjects. To do so, we plan to further refine the study as soon as more data, more buildings and more evacuation drills are available. We encourage the reader to apply this study and to suggest further refinements. We also plan to apply IoT and related technologies: The new indoor location technologies will surely help to better analyze the movements within the building, speeds and slowing zones, helping to further improve this proposal.

## References

[1] Purser D. ASET and RSET: Addressing some issues in relation to occupant behaviour and tenability. Fire Safety Science. 2003;7:91–102. 10.3801/IAFSS.FSS.7-91

[2] Li S, Zhuang J, Shen S. A three-stage evacuation decision-making and behavior model for the onset of an attack. Transportation Research Part C: Emerging Technologies. 2017;79:119–135. 10.1016/j.trc.2017.03.008

[3] Serulle NU, Cirillo C. The optimal time to evacuate: A behavioral dynamic model on Louisiana resident data. Transportation Research part B: Methodological. 2017;106:447–463. 10.1016/j.trb.2017.06.004

[4] Guo RY, Huang HJ, Wong S. Collection, spillback, and dissipation in pedestrian evacuation: A network-based method. Transportation Research part B: Methodological. 2011;45(3):490–506. 10.1016/j.trb.2010.09.009

[5] Kuligowski ED. Burning down the silos: Integrating new perspectives from social science research. In: Proceedings of the 6th International Symposium on Human Behaviour in Fire, Interscience Communications Ltd: London; 2015. p. 1–12.

[6] Ronchi E, Corbetta A, Galea ER, Kinateder M, Kuligowski E, McGrath D, et al New approaches to evacuation modelling for fire safety engineering applications. Fire Safety Journal. 2019;106:197–209. 10.1016/j.firesaf.2019.05.002

[7] Purser DA, Bensilum M. Quantification of behaviour for engineering design standards and escape time calculations. Safety Science. 2001;38(2):157–182. 10.1016/S0925-7535(00)00066-7

[8] Haghani M, Sarvi M. Stated and revealed exit choices of pedestrian crowd evacuees. Transportation Research Part B: Methodological. 2017;95:238–259. 10.1016/j.trb.2016.10.019

[9] Capote J, Alvear D, Abreu O, Cuesta A, Alonso V. A real-time stochastic evacuation model for road tunnels. Safety Science. 2013;52:73–80. 10.1016/j.ssci.2012.02.006

[10] Alvear D, Abreu O, Cuesta A, Alonso V. Decision support system for emergency management: Road tunnels. Tunnelling and Underground Space Technology. 2013;34:13–21. 10.1016/j.tust.2012.10.005

[11] Tavares RM. Evacuation processes versus evacuation models: “Quo Vadimus”? Fire Technology. 2009;45(4):419–430. 10.1007/s10694-008-0063-7

[12] Li D, Han B. Behavioral effect on pedestrian evacuation simulation using cellular automata. Safety Science. 2015;80:41–55. 10.1016/j.ssci.2015.07.003

[13] Lovreglio R, Ronchi E, Borri D. The validation of evacuation simulation models through the analysis of behavioural uncertainty. Reliability Engineering & System Safety. 2014;131:166–174. 10.1016/j.ress.2014.07.007

[14] Hurley MJ, Gottuk DT, Hall JR Jr, Harada K, Kuligowski ED, Puchovsky M, et al SFPE handbook of fire protection engineering. Springer; 2015.

[15] Haghani M, Sarvi M. Crowd behaviour and motion: Empirical methods. Transportation Research Part B: Methodological. 2018;107:253–294. 10.1016/j.trb.2017.06.017

[16] Gibbings JC. Dimensional analysis. Springer Science & Business Media; 2011.

[17] Macagno EO. Historico-critical review of dimensional analysis. Journal of the Franklin Institute. 1971;292(6):391–402. 10.1016/0016-0032(71)90160-8

[18] Vaschy A. Sur les lois de similitude en physique. In: Annales télégraphiques. vol. 19; 1892 p. 25–28.

[19] Buckingham E. On physically similar systems; illustrations of the use of dimensional equations. Physical Review. 1914;4(4):345 10.1103/PhysRev.4.345

[20] Vignaux V, Scott J. Theory & methods: Simplifying regression models using dimensional analysis. Australian & New Zealand Journal of Statistics. 1999;41(1):31–41. 10.1111/1467-842X.00059

[21] Shen W, Davis T, Lin DK, Nachtsheim CJ. Dimensional analysis and its applications in statistics. Journal of Quality Technology. 2014;46(3):185–198. 10.1080/00224065.2014.11917964

[22] Cuesta A, Abreu O, Balboa A, Alvear D. Real-time evacuation route selection methodology for complex buildings. Fire Safety Journal. 2017;91:947–954. 10.1016/j.firesaf.2017.04.011

[23] Tversky A, Kahneman D. Judgment under uncertainty: Heuristics and biases. Science. 1974;185(4157):1124–1131. 10.1126/science.185.4157.1124 17835457

